# The Abscopal Effect: A Review of Pre-Clinical and Clinical Advances

**DOI:** 10.3390/ijms222011061

**Published:** 2021-10-14

**Authors:** James R. Janopaul-Naylor, Yang Shen, David C. Qian, Zachary S. Buchwald

**Affiliations:** Department of Radiation Oncology, Winship Cancer Institute, School of Medicine, Emory University, Atlanta, GA 30322, USA; yang.shen@emory.edu (Y.S.); david.qian@emory.edu (D.C.Q.); zbuchwa@emory.edu (Z.S.B.)

**Keywords:** abscopal effect, radiotherapy, checkpoint blockade

## Abstract

Radiotherapy has been used for more than a hundred years to cure or locally control tumors. Regression of tumors outside of the irradiated field was occasionally observed and is known as the abscopal effect. However, the occurrence of systemic anti-tumor effects was deemed too rare and unpredictable to be a therapeutic goal. Recent studies suggest that immunotherapy and radiation in combination may enhance the abscopal response. Increasing numbers of cases are being reported since the routine implementation of immune checkpoint inhibitors, showing that combined radiotherapy with immunotherapy has a synergistic effect on both local and distant (i.e., unirradiated) tumors. In this review, we summarize pre-clinical and clinical reports, with a specific focus on the mechanisms behind the immunostimulatory effects of radiation and how this is enhanced by immunotherapy.

## 1. Introduction

Photons can be defined by their wavelength, frequency, or energy, with ionizing radiation consisting of photons with at least 10 electron-volts (eV) of energy. Historical sources for ionizing radiation were radioactive elements, which release discrete-energy gamma rays as a result of reactions in the nucleus of an atom, while modern radiation typically uses x-rays created after a high-energy electron interacts with the electric field of an atom. For example, Cobalt-60 (a Cobalt isotope with 33 neutrons in addition to 27 protons) readily decays into Nickel-60, with release of an electron, an anti-neutrino, and two gamma-rays with an energy of 1.17 and 1.33 MeV (average energy of 1.25 MeV). In contrast, modern linear accelerators, also known as LINACs, accelerate electrons to hit a target composed of an element with high number of protons leading to x-rays with energies of 6–18 MeV. Radiation was first used therapeutically in the 1890s for palliation of metastatic gastric cancer and to cure skin cancers [1]. The ionization of DNA either directly from the primary radiation source or secondarily from free radicals generated by interactions of radiation with adjacent water molecules leads to permanent DNA damage that can subsequently lead to apoptosis or mitotic catastrophe when the cell attempts to divide. Since the 1890s, radiation therapy (RT) has continued to provide a role in curative and symptomatic treatment of patients with cancer and benign conditions [2]. Over time, however, there has been an increasing reliance on linear accelerators to deliver RT given better ability to manipulate photon energy and more accurate tumor targeting while avoiding healthy nearby normal tissue. An estimated 50% of all cancer patients will receive some type of RT during their treatment [3]. Traditionally, the effects of RT were thought to be limited to the radiation field alone; however, in 1953, Dr. Mole defined the abscopal effect as “an action at a distance from the irradiated volume” [4]. While the ability of RT alone to act at a distance is rare and typically confined to case reports [5], the combination of RT with modern immunostimulatory therapies has shown promise for patients with a variety of tumors [6], particularly in the metastatic setting. Notably for single or a few sites of disease with poor vasculature, immune-suppressive microenvironment factors, or novel genetic variations that in aggregate decrease the efficacy of systemic therapy, radiation may be able to eradicate those oligo-resistant lesions while preventing transition to potentially less-effective next-line therapy.

**Figure 1:**
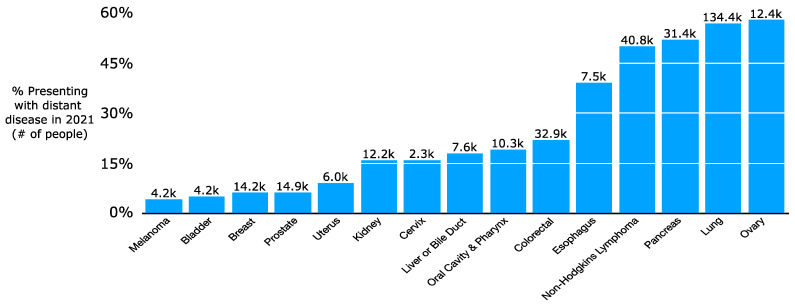
Proportion of patients presenting with upfront distant disease by cancer type in the period 2010–2016.

In 2021, it is projected that more than 300,000 people will be newly diagnosed with upfront metastatic cancer [7]. This does not reflect the additional number of patients initially diagnosed with lower-stage disease who later develop metastatic spread. There is a wide variation in incidence of upfront metastatic disease by histology, with 57% of lung cancer patients but only 6% of breast or prostate cancer patients presenting with distant disease (Figure 1) [8]. While a subset of patients will have a lower systemic burden of metastatic cancer, oligometastatic disease [9], many others will have a high volume of disease. Increasingly, there has been a push for local therapies such as RT or surgery for patients with oligometastatic disease such as a single liver or lung metastasis from colon cancer. There has even been consideration of RT to polymetastatic cancer given promising results with local therapies for oligometastatic disease [10]. The use of local therapies stems in part from the increasing safety of surgery [11,12,13,14,15,16,17] and the ability for RT to be highly conformal with techniques such as Stereotactic Body Radiation Therapy (SBRT) [18,19,20,21]. SBRT is characterized by highly conformal radiation using high-resolution daily imaging (e.g., CT or MRI scan daily for patient setup prior to treatment) in a few fractions, typically 3 to 5, with high doses per fraction. SBRT dosing relies on the fact that the Biologically Effective Dose (BED) required for tumor killing varies based on its sensitivity to radiation; SBRT’s high doses per fraction (e.g., 50 Gy in 5 fractions) can cause similar effective cell kill compared to a more protracted course with higher total dose (e.g., 82 Gy in 41 fractions). Radiation sensitivity is often measured with alpha/beta ratios: high alpha/beta ratios indicate limited repair capability and thus high sensitivity to radiation. Metastatic tumors and rapidly dividing normal tissue (e.g., intestines) have high alpha/beta ratios of 10+ while slow-growing tumors (e.g., soft tissue sarcoma or prostate cancer) or non-dividing normal tissues (e.g., nerves) have low alpha/beta ratios of approximately 1–3. In practice, SBRT in multiple malignancies and resection for up to three liver metastases from colon cancer have been shown on several trials to improve overall or progression-free survival for patients with oligometastatic cancer [22,23,24]. In parallel with the refinement of local therapies in oligometastatic disease, oncologists have made major strides in refining systemic therapy for oligometastatic and polymetastatic cancer. Of particular note, the increasing efficacy and tolerability of immunotherapies for metastatic cancer has accelerated interest in clarifying the role of more definitive intent local therapies for small volume residual or progressive metastases [25,26,27,28,29]. 

Typically, the anti-cancer immune response is mediated, in part, by dendritic cells (DCs) presenting tumor neoantigens to T cells. To evade this response, tumors may impair T cell activity and DC-mediated stimulation through inhibitory signals including upregulation of programmed death ligand 1 (PD-L1), which has been seen in up to 50% of cancer patients [30]. The use of T cell immune checkpoint inhibitors (ICI) to overcome cancer-mediated T cell inhibition including anti-PD-1, anti-PD-L1 and anti-CTLA-4 monoclonal antibodies has shown particular promise in patients with metastatic cancer [31,32,33,34], with encouraging results in animal models and clinical trials. While the results with these ICI in melanoma and lung cancers in clinical trials showed a dramatic increase in overall survival for patients with metastatic or even locally advanced disease [32,35,36], they have also proven to be effective in primary tumors that are less immunogenic including liver cancers [25]. However, many patients do not initially respond to ICI and so additional therapies to enhance the anti-tumor immune response have been tested including dual-ICI and direct tumor killing therapies that may help enhance antigen presentation to stimulate the T cell response. Spurred by pre-clinical and clinical reports of RT improving response to ICI [37], the combination of RT and ICI has become a new and very active area of research for the metastatic patient population. Both mechanisms and the magnitude of maximal potential benefit were unclear until recently, particularly since RT was initially considered to be an inhibitor of the immune response through lymphodepletion rather than an immune stimulator. In recent years, however, new prospective studies and cases in humans have been reported, helping to clarify optimal patient selection [38,39], sequencing [40], targets [40,41], and possible limitations [42,43,44] for RT with ICI. Despite this progress, much work is still needed given the diversity of tumors, presentations, and immune mechanisms involved.

## 2. Immunostimulation by Radiotherapy Alone and the Abscopal Effect

### 2.1. Pre-Clinical Data

**Figure 2:**
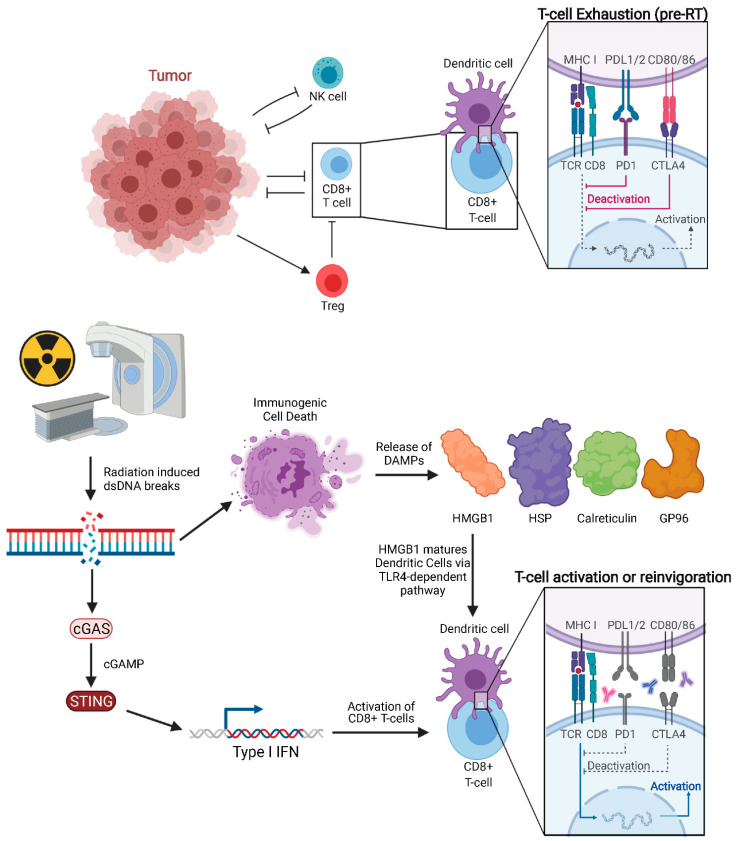
Pre-treated tumor with exhausted CD8^+^ T cells and tumor proliferating without immune inhibition. Radiation of tumors leads to double-stranded DNA breaks and downstream cGAS-STING signaling, which in turn increases Type 1 IFN release. Immunogenic cell death releases DAMPs such as HMGB1, HSP, GP96, and calreticulin. HMGB1 activates Dendritic Cells through TLR4-dependent pathway. Anti-CTLA-4 agents act on naïve and regulatory T cells while anti-PD-1 agents predominantly work on exhausted T cells.

Classically, RT was considered to be immunosuppressive, as lymphocytes are among the most radiosensitive cells. Lymphocytopenia can be commonly seen in total body irradiation prior to stem cell transplant or craniospinal irradiation given that lymphocyte depletion from RT is dependent on volume of tissue irradiated. Despite this, many studies, both pre-clinical and clinical, support the hypothesis that a spontaneous regression of tumors outside of the irradiated field is mediated by the immune system [45,46,47]. In 2004, Demaria reported that abscopal effects induced by RT are immune-mediated as abscopal tumor regression was not observed in immunodeficient mice [48]. This was among the earlier studies which explicitly drew this conclusion. Pre-clinical models have now investigated RT′s mechanism of immune-stimulation, showing that RT can enhance the antigen-processing and presentation pathway [45,46,47], particularly major histocompatibility complex (MHC) class I levels [49]. The main effectors of tumor cell killing are CD8^+^ T cells (cytotoxic T lymphocytes, CTL), which rely on neoantigen presentation by DCs. Notably, RT-induced cell death facilitates neoantigen cross-presentation in DCs and CD8^+^ T cell stimulation by activating Toll-Like Receptor 4 (TLR4) and type I interferon (IFN) signaling (Figure 2) [50,51]. Rodriguez-Ruiz et al. reported that abscopal effects of RT are dependent on CD8^+^ T cells and cross-priming DCs [52]. However, to access DCs and tumor antigens, CD8^+^ T cells may rely on intercellular adhesion molecule-1 (ICAM-1) to facilitate tumor infiltration. Animal models have shown that ICAM-1 concentration on PET imaging with Copper-64-labeled probes correlates negatively with growth of non-irradiated tumors, indicating a potential role in the abscopal effect [53]. 

Damage-associated molecular patterns (DAMPs) are released from cells following necrosis. Recent studies suggest that specific forms of programmed cell death such as necroptosis and immunogenic cell death (ICD) following RT can also trigger release of DAMPs into the extracellular space, including high-motility group box 1 (HMGB1), heat shock proteins (HSP), calreticulin membrane exposure, Glucose regulated protein 96 (GP96), and surface-exposed calreticulin [54,55,56,57]. Of note, other local therapies, such as irreversible electroporation in orthotopic pancreatic cancer models have been shown to trigger immunogenic cell death with subsequent secretion of DAMPs [58]. HMGB1 is one of the most abundant danger signals released following RT [59]. Calreticulin, a highly conserved endoplasmic reticulum chaperone protein, can stimulate phagocytosis of cancer cells by DC [60], while HMGB1, a critical chromatin protein, promotes antigen presentation [61]. HMGB1 release triggered by RT enhances DC-mediated antigen presentation through TLR4-dependent signaling pathway [50]. This in turn matures DCs [60] and stimulates subsequent T cell-mediated tumor lysis [62]. Innate immunity can also work independently and in tandem with the adaptive anti-tumor immune response. For example, human, but not murine, neutrophils can release catalytically active neutrophil elastase to specifically kill cancer cells and liberate the CD95 death domain, helping to enhance CD8^+^ T cell-mediated abscopal effects [63]. 

RT is a well-documented inducer of DNA damage, which is an important underlying mechanism for abscopal effect initiation. RT-induced DNA damage occurs mainly through: (1) direct breakage of DNA by high-energy secondary electrons and (2) the generation of free radicals [64,65]. RT-induced double-strand breaks (DSBs) are the most lethal type of DNA damage. DSBs leads to the formation of micronuclei, whose defective membrane can expose double-stranded DNA (dsDNA) to the cytosolic dsDNA sensor cyclic GMP-AMP synthase (cGAS). cGAS is a pattern recognition receptor that triggers type I IFN production through the downstream stimulator of IFN genes (STING). The importance of the cGAS-STING pathway on the anti-tumor immune response stimulated by both RT and anti-PD1/L1 has now been established. The ability of RT to efficiently induce the abscopal effect depends on type I IFN secreted by irradiated cells. Mitochondrial outer membrane permeabilization (MOMP), a key step in apoptosis, is known to drive robust type I IFN secretion. This secretion is more pronounced when mitochondrial autophagy is disabled, or apoptotic caspases are inactivated as is typical of cancer cells. Yamazaki et al. demonstrated that autophagy inhibits RT-driven cGAS-dependent type I IFN secretion secondary to the cytosolic accumulation of mitochondrial DNA (mtDNA) in mouse cancer cells [66]. Furthermore, autophagy inhibition promotes type I IFN–dependent RT abscopal effects. It has also been shown that the STING signaling pathway is activated in DCs, and cGAS is essential for the sensing by the DC of irradiated-tumor cell derived dsDNA. They also demonstrated that STING promotes an anti-tumor CD8^+^ T cell response with an increased frequency of IFN-*γ*^+^ CD8^+^ T cells in the tumor-draining lymph node (TDLN).

The importance of the TDLN in mediating a robust, RT-stimulated, anti-tumor response and RT′s synergy with ICI has recently been reported, but only begun to be characterized [67,68]. The TDLN plays a critical role in T cell stimulation through activating DCs and concentrating tumor antigens [67,69,70]. Furthermore, there appears to be a progenitor sub-population of CD8^+^ T cells both within the tumor and TDLN that is critical for robust PD-1 therapy responses [71,72]. While tumor-directed RT increases progenitor T cells in the distant, non-irradiated tumor, this effect was attenuated by TDLN-directed RT [67]. This enhancement of the progenitor T cell density in non-irradiated tumors is a potential mechanism for the synergy between RT and ICI which is discussed in the pages that follow. Additionally, given the proximity of TDLN to typical tumor-directed RT and historical concern for TDLN as site for cancer spread, there is a dearth of clinical data about RT designed to avoid TDLN. However, further pre-clinical work will help elucidate mechanisms as human studies are being designed and carried out.

In contrast to a few fractions of SBRT, low-dose fractionated RT (e.g., ≤3 Gy per fraction to ≤30 Gy total dose) is not immunostimulatory. Reijmen et al. evaluated the therapeutic and immunologic effects of low-dose fractionated RT on lung cancers in mice. They found that 4 consecutive daily fractions of RT at 3.2 Gy severely reduced the number of CD8^+^ T cells and mature antigen presenting cells within lung tumors [73]. Therefore, the ideal use of radiation as an immune stimulator may be induction of immunogenic cell death of tumor cells while avoiding radiation to the TDLN to preserve populations of CD8^+^ T cells and mature antigen presenting cells.

Notably, in vitro and in vivo systems have demonstrated both dose and timing relationships between RT and the immune response [74]. Further work is being done to characterize these relationships, though mostly in the setting of ICI, which has shown more consistent and frequent systemic responses. Awareness that induction of anti-tumor immune responses, apart from RT′s direct tumoricidal activity, has led to increased clinical interest in RT′s immunostimulatory activities. Additionally, avoidance of the TDLN and highly conformal RT techniques to avoid lymphocytopenia appear to be critical to optimizing the immunostimulatory effects of RT.

### 2.2. Clinical Studies

The abscopal effect in patients with metastatic cancer treated with radiation alone has been documented although it is a rare occurrence. A systematic review showed that between 1969 and 2014, there were 46 published case reports with non-irradiated, distant response typically occurring 2 months after radiation [5]. Notably there have been case reports in multiple different primary tumors from melanoma [75] to cholangiocarcinoma [39] to renal cell carcinoma [76]. In one patient with NSCLC on Osimertinib monotherapy, whole-brain radiation led to a 3 cm shrinkage of her primary lesion. After subsequently being treated with chemotherapy followed by atezolizumab, she had abscopal response again in lung primary following palliative radiation to bony metastasis [77]. Work in animal models, however, suggests a correlation between total dose and rates of abscopal responses with BED 60 Gy for tumors with standard alpha/beta ratio estimate of 10 was associated with up to 50% out-of-field responses [78]. A BED 60 Gy corresponds to SBRT courses of 35 Gy in 5 fractions, while commonly used doses such as 24 Gy in 3 fractions, 30 Gy in 5 fractions, and 27 Gy in 3 fractions correspond to BED 43.2 Gy, 48 Gy, and 51.3 Gy, respectively. Of note, higher doses to surrounding normal tissues, particularly with few fractions as is typical with SBRT, can greatly increase risk of late complications for long-term survivors. The luminal gastrointestinal structures and kidneys are particularly sensitive to high doses per fraction and high total doses of radiation.

Preferentially targeting hypoxic tumor areas may be an important immune-stimulatory approach given their role in immunosuppression [79,80]. Additionally, limiting the target volume could allow better sparing of TDLNs and decrease risk of lymphodepletion. Promising results from Austria show that targeting hypoxic tumor cores alone with hypofractionated or ablative radiotherapy provide improved local control, disease-free survival, and cancer-specific survival [41,81]. For 60 patients with non-small-cell lung cancer (NSCLC) who were not candidates for standard of care chemoradiation due to size of locoregional disease, Tubin et al. non-randomly assigned them to systemic therapy (*n* = 20), palliative RT (3 Gy × 10 fractions, *n* = 20) or SBRT (*n* = 20) to the hypoxic core of the primary tumor alone. The target included the non-enhancing portion of the primary tumor on contrast-enhanced CT scan (defined as the anoxic core) treated to 10–12 Gy to the 70% isodose line with an attempt to limit dose to peritumoral area (defined as 1 cm from anoxic core). Patients could receive up to two additional doses of 10 Gy in 1 fraction delivered after one-month restaging. With median follow-up of 13 months, one-year overall survival was 75% and 60% in the SBRT and systemic therapy groups, respectively, (*p* = 0.09) with one-year progression-free survival of 60% and 15% (*p* = 0.003) and cancer-specific survival of 90% and 60% (*p* = 0.049), respectively [41]. 

Recently reported data from the TARGIT-A trial, a risk-adapted targeted intraoperative radiotherapy (TARGIT-IORT) during lumpectomy for breast cancer, showed IORT to be as effective as whole-breast external beam radiotherapy (EBRT) [82]. Vaidya et al. presented further detailed analyses of the trial and concluded that TARGIT-IORT is as effective as EBRT in all subgroups for local recurrence-free survival (HR 0.75; 95% CI 0.57–1.003; *p* = 0.052) and overall survival (HR 0.96; 95% CI 0.68–1.35; *p* = 0.80) [83]. One of the striking findings was that unlike EBRT where local recurrence was a powerful predictor of distant metastases, breast cancer mortality and overall mortality, local recurrence following IORT did not have worse survival outcomes, suggesting possible action at a distance. Additionally, a significant reduction in non-breast cancer mortality in patients randomized to TARGIT-IORT (HR 0.38; 95% CI 0.17–0.88; *p* = 0.0093) was noted, which the authors speculate may be secondary to a global abscopal effect suppressing other malignancies.

## 3. Combined Radiation with ICI and the Abscopal Effect

### 3.1. Pre-Clinical Data

The mechanism by which RT enhances ICI is thought to be either or both reinvigoration of exhausted intra-tumoral CD8^+^ T cells or proliferation and differentiation of naïve T cells [67,84]. Immunostimulatory monoclonal antibodies (mAb) have already been combined with RT in pre-clinical models. Studies on anti-CTLA-4 mAb, anti-PD-1 mAb, and anti-CD137 mAb showed synergistic effects with RT. Furthermore, RT in combination with anti-CTLA-4 and anti-PD-1 produced an abscopal effect [49,84,85,86,87]. While CTLA-4 antagonists primarily act on naïve and regulatory T cells [88], anti-PD-1 agents work on newly activated and exhausted T cells [89]. 

Pre-clinical work shows that different animal models, ICI agents, and variations in RT lead to variable responses. Optimal RT and immunotherapy sequencing may also depend on the immunomodulatory agent utilized. For example, in a mouse melanoma model, 12 Gy compared to 5 Gy delivered on two sequential days led to peak CD8^+^ T cell expansion at 5 days versus 8 days post-RT followed by gradual versus more rapid decline, respectively [90]. With anti-PD-L1 agents, optimal outcomes were seen when RT was given concurrently [91]. However, with CTLA-4 antagonists the best results were observed with RT delivered after ICI, and with anti-OX40, a co-stimulatory molecule, when RT was delivered 1 day prior [92]. Murakami et al. showed that combination IL-15, a potent stimulator of both NK T cells and CD8^+^ T cells, and that RT elicited an anti-tumoral immune response which was further optimized by DTA-1, a glucocorticoid-induced tumor necrosis factor receptor–related protein (GITR) agonist [93]. One elegant study by Wei et al. demonstrated that the sequencing of anti-PD-1 administration relative to RT determined the magnitude of the abscopal response. Anti-PD-1 administration after local tumor RT resulted in the expansion of polyfunctional intratumoral CD8^+^ T cells, a decrease in intratumoral dysfunctional CD8^+^ T cells, expansion of reprogrammable CD8^+^ T cells, and induction of potent abscopal responses. However, administration of anti-PD-1 before RT resulted in increased CD8^+^ T cell radiosensitivity leading to CD8^+^ T cell programmed cell death. The subsequent increase in apoptotic intratumoral CD8^+^ T cells delayed the expansion of effector CD8^+^ T cells and almost completely abrogated systemic immunity [94]. This study provides robust pre-clinical evidence for the importance of optimizing the timing of immunotherapeutic administration when designing clinical trials examining combination RT and immunotherapy.

### 3.2. Clinical Evidence

Results in humans have been promising in the sequential setting with either CTLA-4 antagonists [40] or PD-L1 inhibitors [35]. The largest prospective, randomized study assessing ICI addition to RT in patients was the PACIFIC trial (NCT02125461), showing an overall survival improvement for patients with locally advanced NSCLC treated with adjuvant durvalumab (anti-PD-L1) after definitive intent fractionated chemoradiation (HR 0.68; 99.73% CI 0.47–0.997; *p* = 0.0025) [35]. This changed the standard of care. Recently, CheckMate 577 (NCT02743494), a phase 3 prospective, randomized trial, showed that the addition of adjuvant nivolumab following chemoradiation and surgery for locally advanced esophageal cancer more than doubles median disease-free survival (11.0 vs. 22.4 months, HR 0.69; 95% CI 0.56–0.86; *p* < 0.001) [95]. Both PACIFIC and CheckMate 577 added immunotherapy after completion of radiation. However, the optimal sequencing, particularly with distinct agents, is an ongoing area of research. Other combinations of different histologies with either fractionated or hypofractionated RT with ipilimumab [38,40,96,97], nivolumab [42,98], pembrolizumab [44], GM-CSF [99], TGF-beta blockade [43], intratumoral dendritic cell injection [100], or PV-10 [101] have shown mixed results.

Using the CTLA-4 inhibitor ipilimumab, Welsh et al. showed in 106 patients with liver or lung metastases treated with 50 Gy in 4 fractions or 60 Gy in 10 fractions with either the first or second cycle of ICI can lead to significant responses in non-irradiated lesions. They found that up to 42% of patients with lung metastases targeted after completion of 1st cycle IO had abscopal responses with higher response rates in adjacent lesions receiving incidental low-dose RT [40]. However, there was no statistically significant difference in non-irradiated lesion response with RT delivered concurrent with the 1st cycle of ICI (20%) compared to sequential RT around the time of the 2nd cycle of ICI (28%). In a smaller study of 39 patients with metastatic NSCLC, RT delivered to 27 Gy in 3 fractions or 30 Gy in 5 fractions combined with ipilimumab had an 18% response rate in non-irradiated lesions though 2 patients (5%) had complete response [38]. Of note, ipilimumab was given concurrently in both of these trials. One of the major concerns with use of local therapies in patients with metastatic disease is time off systemic therapy. While concurrent ICI can allay those fears, sequential RT and ICI has been more successful in both small and large clinical trials.

In the PEMBRO-RT (NCT02492568) phase 2 prospective, randomized trial, SBRT administration prior to pembrolizumab resulted in a doubling of the response rate in patients with metastatic NSCLC. The overall response rate at 12 weeks was 18% in the control arm vs. 36% in the experimental arm. The PEMBRO-RT study is the first randomized trial to show an augmenting effect of SBRT on the response to PD-1 blockade in patients with metastatic NSCLC though was not powered to show a survival benefit (HR 0.66; 95% CI 0.37–1.18; *p* = 0.16). The results of this study are encouraging and further evaluation in a larger phase 2/3 trial is recommended to confirm the findings [102]. Luke et al., in a single-arm trial, showed that pembrolizumab initiated within 7 days after SBRT to two to four progressing metastases yielded a non-irradiated response rate of 26.9% with six (8.2%) Grade 3 toxicities. Exploratory analysis of pre- and post-SBRT tumor biopsies showed changes in IFN gene expression after SBRT, but was limited by sample size [103]. While over one-third of patients may respond to SBRT followed by pembrolizumab, further work is needed to see if these improvements are both clinically and statistically significant in larger populations and if further dosing and timing adjustments could raise response rates even more. 

In head and neck cancer (HNC), the data have been more equivocal. In a prospective, randomized phase 2 trial, 62 patients with metastatic squamous cell HNC treated with nivolumab with or without concurrent SBRT to 27 Gy in 3 fractions between the first and second cycle of nivolumab. There was no difference in non-irradiated lesion response with SBRT (34%) compared to nivolumab alone (29%) [42]. The study indicated that the abscopal effect remains rare and may require more careful patient selection when using anti-PD-1/PD-L1 therapies and SBRT for HNC. However, in contrast to the PEMBRO-RT trial, the ICI was delivered concurrent with SBRT as opposed to sequentially after the SBRT. In the JAVELIN head & neck 100 (NCT02952586) phase 3 randomized, double-blind, placebo-controlled study of chemoradiation with or without concurrent avelumab (anti-PD-1) for locally advanced squamous cell HNC, there was no significant benefit for the 350 patients randomized to avelumab compared to the 347 randomized to placebo albeit minimal differences in toxicity (HR 1.21; 95% CI 0.93–1.57; *p* = 0.92) [104]. In a single-arm study of 21 patients treated with concurrent SBRT and nivolumab followed by definitive surgical resection and 3 months of adjuvant nivolumab, the pathologic complete response rate was 67% with tolerable toxicity [105]. As opposed to traditional radiation for locally advanced squamous cell HNC, irradiating tumor expansions and elective lymph node basins, the SBRT delivered was to gross tumor alone in either 40 Gy in 5 fractions or 24 Gy in 3 fractions. Similar to the results in head and neck cancers, a single-arm, phase 2 trial in refractory extra-medullary multiple myeloma closed early after only 4 patients enrolled due to the pandemic, and showed minimal benefit for combination of RT with cycle 2 of avelumab [106]. These studies highlight the persistent challenges of differences in histology, patient selection, radiation technique, and optimal timing of immunotherapy with other treatments.

Notably, some caution should be taken when combining SBRT with immunotherapy in certain parts of the body. The PLUMMB Trial (NCT02560636), a phase 1 trial of muscle invasive or metastatic bladder cancer treated with pembrolizumab and SBRT to 36 Gy in 6 fractions, was stopped early after toxicity in the first five patients. Three patients had Grade 3 urinary toxicity and one patient had a Grade 4 bowel perforation [44]. These are much higher rates of high grade toxicity than expected with definitive intent chemoradiation. For muscle invasive bladder cancer with conventionally fractionated or moderately hypofractionated RT late Grade 3–4 toxicity range from 8% to 16% [107,108]. In contrast to unacceptably high gastrointestinal and genitourinary toxicity with bladder SBRT and ICI, the skin provides an excellent location for safe radiation dosing. SBRT to 24 Gy in 3 fractions between the 1st and 2nd cycles of nivolumab for 20 patients with inoperable or metastatic cutaneous melanoma was tolerable and had a 45% response rate in non-irradiated lesions. Furthermore, circulating tumor DNA was an accurate predictor of response and progression following treatment [98]. Melanoma continues to be an excellent target for ICI and RT given the high immunogenicity of tumors even in patients with unfavorable pre-treatment immune signatures [109], while the lack of response with squamous cell tumors and concerning toxicity with SBRT in the pelvis requires further investigation of safe immune-stimulatory RT regimens.

Combinations of RT with other immunomodulatory agents have shown promising early signals. In 41 patients with metastatic solid tumors refractory to systemic therapy, 35 Gy in 10 fractions combined with granulocyte-macrophage colony-stimulating factor triggered an abscopal response in 11 patients (27%) [99]. In a randomized study of 23 patients with metastatic breast cancer, use of higher doses of fresolimumab, a TGF-beta antagonist, with SBRT to 22.5 Gy in 3 fractions was associated with higher median overall survival (HR 2.73; 95% CI 1.02–7.30; *p* = 0.039,) with the higher dose correlating with a boost in CD8^+^ T cells. However, 30% of patients had a Grade 3 or 4 toxicity [43]. While toxicity remains a concern for any novel agent, the continued investigation and translation of pre-clinical work to humans should be encouraged.

Intra-lesional injections combined with RT have been less studied. In a trial of 15 patients with metastatic solid tumors receiving cyclophosphamide followed by intratumoral dendritic cell injection, 6 patients had RT with 1 of the 6 patients having an abscopal response in lungs and upper abdomen following pelvic radiotherapy, while 5 of 6 patients receiving RT had stable disease and reduction in concerning lesions on PET scan [100]. Using intralesional PV-10 alone, a non-pyrogenic 10% solution of Rose Bengal, has led to overall response rates of approximately one-third, but when combined with RT to 30 Gy in 6 fractions delivered twice weekly, the response rates increased to 86% with a 33% complete response rate for 15 patients [101]. 

Based on these data, the study of optimal radiation doses, fractionation schemes, and combinations with ICI offers a promising approach for improving the efficacy of cancer treatments. While caution should be taken with invasive surgeries or SBRT in sensitive areas such as the pelvis, the use of local therapy has shown significant promise for patients with metastatic cancer. Combinations of RT appear to have significant immunostimulatory effect when radiation fields are optimized to induce immunogenic cell death in tumor while preserving TDLN and preventing excessive lymphodepletion. Further work is needed to harmonize maximal immunostimulatory effects of RT with risks of systemic progression off ICI or other systemic therapy.

## 4. Future Directions

While the most promising results with RT and ICI have come in patients with melanoma and NSCLC, further work enhancing response rates with other histologies is needed. Recent studies have shown no significant benefit for squamous cell HNC [42,104], potential dangers and toxicities that can come with SBRT and ICI in bladder cancer [44], but promising results in NSCLC with SBRT alone targeting hypoxic areas [41] and with conventional radiation followed by durvalumab [35]. Optimal sequencing, dosing, and tolerability with different ICI agents remains the subject of active investigation. 

There are multiple open protocols looking at different combinations of RT and ICI, particularly for thoracic malignancies (Table 1). Following the significant benefit of IO added to standard-of-care chemotherapy for extensive-stage small-cell lung cancer seen in two prospective, phase 3 randomized trials—IMPower 133 (NCT02763579) [110] and CASPIAN (NCT03043872) [36]—NRG LU-007, also known as the RAPTOR Trial (NCT04402788), is a prospective, multi-center phase 3 trial that will examine the role of consolidation radiation for these patients receiving the newly defined standard of chemotherapy and ICI for extensive-stage small-cell lung cancer [111]. In contrast, NRG LU-005 (NCT03811002) is a phase 3, randomized trial examining the role of ICI added to standard-of-care chemotherapy and radiation for limited-stage small-cell lung cancer [112]. The FORCE trial (NCT3044626), a prospective, open-label, non-randomized phase 2 trial, and RTOG 3505 (NCT02768558), a prospective, phase 3, randomized trial, examine the combination of ICI and RT for patients with advanced (i.e., metastatic) and locally advanced NSCLC, respectively [113,114]. PACIFIC-4 (NCT03833154) builds off the survival benefit of durvalumab with chemoradiation for locally advanced NSCLC seen in the original PACIFIC trial [35] by examining the role of durvalumab in the management of patients with early NSCLC treated with SBRT with a phase 3, prospective, placebo-controlled, randomized trial design [115]. The Radio-Immunotherapy before cystectomy in locally advanced urothelial carcinoma of the bladder (RACE IT) trial (NCT03529890) plans to evaluate the role of neoadjuvant ICI and RT prior to definitive surgery for locally advanced bladder cancer in a single-arm phase 2 design [116], while the PLUMMB trial, examining SBRT and immunotherapy for muscle invasive or metastatic bladder cancer, is being revised given toxicity concerns to use lower doses of RT [44]. The PRIMMO study (NCT03192059) is an open-label, non-randomized, 3-cohort phase 2 trial examining the outcomes from chemotherapy followed by concurrent pembrolizumab with SBRT (24 Gy in 3 fractions) in patients with recurrent or refractory cervical carcinoma, endometrial carcinoma, or uterine sarcoma [117]. These trials will enhance our understanding of both universal principles in combining ICI and RT and site-specific factors that may modulate local and abscopal effects. Additionally pre-clinical results such as ICAM-1-specific PET imaging may provide future patients with prognostic and predictive data to inform care [53], or novel techniques such as electroporation may help overcome the challenges of the cancer microenvironment [58]. 

## Figures and Tables

**Table 1:** A selection of ongoing clinical trials evaluating abscopal responses and/or combination radiation and immunotherapy. NSCLC = non- small-cell lung cancer, RCC = renal cell carcinoma, and H&N SCC = head and neck squamous cell carcinoma.

Type of Cancer	Irradiation Regimen	Immunotherapy	Sequence of Therapies	Clinical Trial Number	Start Date	Phase	Accrual Goal
Colorectal Cancer with Liver Metastases	Yttrium-90 Radioembolization	Durvalumab	IO pre- and post-radiation	NCT04108481	10/1/21	1/2	18
NSCLC	30–50 Gy in/5 Fractions (2–4 lesions)	Toripalimab/Bevacizumab	IO with and post-radiation	NCT04238169	9/1/20	2	60
NSCLC	20 × 2 Gy (daily over 4 weeks) or 5 × 5 Gy (daily over 1 week) or 3 × 8 Gy (on alternate days over 1 week)	Durvalumab	Chemo then IO pre- and with radiation followed by surgery and post-op IO	NCT04245514	7/1/20	2	90
Classical Hodgkin Lymphoma	1 × 20Gy	Nivolumab	IO with and post-radiation	NCT03480334	12/1/19	2	29
NSCLC, RCC, H&N SCC, and Melanoma	8 Gy × 3 fractions	Pembrolizumab and intralesional IL-2	Radiation with 2nd of 4 cycles IO	NCT03474497	5/1/19	1/2	45
Unknown Primary	20–30 Gy over five fractions for up to two cycles	Pembrolizumab	Radiation with 2nd cycle of up to 24 months of IO	NCT03396471	2/1/18	2	34
NSCLC	Image-guided radiation therapy	Nivolumab/Pembrolizumab/Atezolizumab	Radiation within 2 weeks of patient′s standard-of-care IO	NCT03176173	6/1/17	2	85
Mesothelioma	3–5 fractions of SBRT	Immunotherapy at discretion of medical oncology	IO at discretion of medical oncologist	NCT04926948	6/1/21	1	20
NSCLC	SBRT	IL-19-IL-2	IO post-radiation only	NCT03705403 IMMUNOSABR2	4/1/19	2	126
